# Increasing incidence of thyroid cancer in the Nordic countries with main focus on Swedish data

**DOI:** 10.1186/s12885-016-2429-4

**Published:** 2016-07-07

**Authors:** Michael Carlberg, Lena Hedendahl, Mikko Ahonen, Tarmo Koppel, Lennart Hardell

**Affiliations:** Department of Oncology, Faculty of Medicine and Health, Örebro University, SE-701 82 Örebro, Sweden; Independent Environment and Health Research Luleå, Östra Skolgatan 12, SE-972 53 Luleå, Sweden; Institute of Environmental Health and Safety, Jaama 14-3, 11615 Tallinn, Estonia; Department of Labour Environment and Safety, Tallinn University of Technology, SCO351 Ehitajate tee 5, 19086 Tallinn, Estonia

**Keywords:** Mobile phone, Cordless phone, Thyroid cancer, Swedish Cancer Register, NORDCAN, Radiofrequency electromagnetic fields, RF-EMF, Ionizing radiation, Incidence, Nordic countries

## Abstract

**Background:**

Radiofrequency radiation in the frequency range 30 kHz–300 GHz was evaluated to be Group 2B, i.e. ‘possibly’ carcinogenic to humans, by the International Agency for Research on Cancer (IARC) at WHO in May 2011. Among the evaluated devices were mobile and cordless phones, since they emit radiofrequency electromagnetic fields (RF-EMF). In addition to the brain, another organ, the thyroid gland, also receives high exposure. The incidence of thyroid cancer is increasing in many countries, especially the papillary type that is the most radiosensitive type.

**Methods:**

We used the Swedish Cancer Register to study the incidence of thyroid cancer during 1970–2013 using joinpoint regression analysis.

**Results:**

In women, the incidence increased statistically significantly during the whole study period; average annual percentage change (AAPC) +1.19 % (95 % confidence interval (CI) +0.56, +1.83 %). Two joinpoints were detected, 1979 and 2001, with a high increase of the incidence during the last period 2001–2013 with an annual percentage change (APC) of +5.34 % (95 % CI +3.93, +6.77 %). AAPC for all men during 1970–2013 was +0.77 % (95 % CI −0.03, +1.58 %). One joinpoint was detected in 2005 with a statistically significant increase in incidence during 2005–2013; APC +7.56 % (95 % CI +3.34, +11.96 %). Based on NORDCAN data, there was a statistically significant increase in the incidence of thyroid cancer in the Nordic countries during the same time period. In both women and men a joinpoint was detected in 2006. The incidence increased during 2006–2013 in women; APC +6.16 % (95 % CI +3.94, +8.42 %) and in men; APC +6.84 % (95 % CI +3.69, +10.08 %), thus showing similar results as the Swedish Cancer Register. Analyses based on data from the Cancer Register showed that the increasing trend in Sweden was mainly caused by thyroid cancer of the papillary type.

**Conclusions:**

We postulate that the whole increase cannot be attributed to better diagnostic procedures. Increasing exposure to ionizing radiation, e.g. medical computed tomography (CT) scans, and to RF-EMF (non-ionizing radiation) should be further studied. The design of our study does not permit conclusions regarding causality.

## Background

Thyroid cancer is a relatively rare cancer. In total, 157 men and 429 women were reported to the Swedish Cancer Register in 2013, or 0.95 % of all cancer cases [1]. It is two to three times more common in women, although the proportion is affected by age and histologic type [2]. Reproductive and hormonal factors have been suggested to explain this gender difference [3, 4]. Ionizing radiation was first suggested in the late 1940s and early 1950s to be associated with an increased risk for thyroid cancer [5, 6]. It is the only well-established risk factor as shown for external radiotherapy [7, 8], diagnostic X-ray investigations [9], among A-bomb survivors in Hiroshima and Nagasaki [10] and after the Chernobyl and Fukushima disasters [11–13].

Papillary thyroid cancer is the most common histologic type and represents 60–70 % of all cancers. It has the best prognosis with 10-year survival rates varying between 60 and 95 % [14, 15]. The papillary type is also the most common radiation induced thyroid cancer [16]. The follicular type occurs in about 20 % of all thyroid cancer cases. The prognosis is somewhat worse than for the papillary type [15, 17]. There is also a mixed papillary-follicular type, usually classified as papillary thyroid cancer. The medullary type represents 4–10 % of all thyroid cancer cases and is usually sporadic or familial [18]. The anaplastic thyroid cancer is an aggressive type representing about 10 % of all thyroid cancer cases. It affects mainly elderly patients and the median survival time has been reported to be in the range of 3 to 6 months [19, 20].

The generally good prognosis for survival makes studies on incident cases more preferable than using mortality data. The aim of this study was to use the Swedish Cancer Register to study the incidence of thyroid cancer. In the diagnostic procedure, histology and/or cytology are usually included. Due to the anatomical localization it is easy to get a specimen for examination. It is compulsory for all health care providers to report new diagnostic cancer cases to the register and most pathology departments have routines for doing so. Thus, the Swedish Cancer Register was used for this study based on official data without any personal identification. Approval by the ethical committee was not necessary.

## Methods

### Study design

The National Board of Health and Welfare administers the Swedish Cancer Register which was started in 1958. The basis for diagnosis can be clinical examination, histology/cytology, surgery, autopsy, or other examinations such as computed tomography (CT)/magnetic resonance imaging (MRI) or laboratory investigations. Incidence per 100,000 person-years, age-adjusted according to the world population, was analyzed for the ICD-7 code 194, i.e. thyroid cancer based on data in the Swedish Cancer Register for the time period 1970–2013. This data is available online (http://www.socialstyrelsen.se/statistik/statistikdatabas/cancer).

To study the incidence of different types of thyroid cancer, data was obtained from the Swedish Cancer Register for the time period 1993–2013 (earlier data is not available). Due to low numbers of cases with rare types of thyroid cancer a wider age group was used for the youngest group, 0–39 years instead of 0–19 and 20–39 years as was used for thyroid cancer in total.

In addition we used NORDCAN to assess incidence data (ICD-10 code C73 = thyroid cancer) for all Nordic countries (available at http://www-dep.iarc.fr/NORDCAN/english/frame.asp). This data (age-adjusted according to the world population) was retrieved for the same time period as from the Swedish Cancer Register, 1970 to 2013, and included Sweden, Denmark, Finland, Norway and Iceland.

### Statistical methods

The NCI Joinpoint Regression Analysis program, version 4.1.1.1 was used to examine trends in age-standardized incidence by fitting a model of 0–4 joinpoints using settings in default mode [21]. When joinpoints were detected, annual percentage change (APC) and 95 % CIs were calculated for each linear segment. Average annual percentage changes (AAPC) were also calculated for the whole time period using the average of the APCs weighted by the length of the segment.

## Results

### The Swedish Cancer Register

In women the incidence increased statistically significantly during the whole study period 1970–2013; AAPC +1.19 % (95 % CI +0.56, +1.83 %). Two joinpoints were detected, 1979 and 2001; 1970–1979 APC +2.15 % (95 % CI +0.05, +4.30 %); 1979–2001 APC −1.39 % (95 % CI −1.96, −0.82 %); 2001–2013 APC +5.34 % (95 % CI +3.93, +6.77 %), see Table 1. In the age group 0–19 years no joinpoint was found, but the incidence increased throughout the period with an AAPC of +1.32 % (95 % CI +0.41, +2.24 %). In the age group 20–39 years one joinpoint was detected in 2006, with a high APC for the time period 2006–2013; +10.77 % (95 % CI +5.75, +16.04 %). That age group also showed the highest AAPC for the whole study period; AAPC +2.27 % (95 % CI +1.46, +3.09 %). For 40–59 year old women, one joinpoint was found in 2001 with a statistically significant increase in incidence during 2001–2013; APC +5.03 % (95 % CI +2.02, +8.13 %). Women aged 60–79 years showed a statistically significant increase in incidence during 2004–2013; APC +6.90 % (95 % CI +3.71, +10.19 %).

**Table 1:** Joinpoint regression analysis of thyroid cancer incidence in women in the Swedish Cancer Register

ICD-7	Joinpoint location	APC 1 (95 % CI)	APC 2 (95 % CI)	APC 3 (95 % CI)	AAPC (95 % CI)
194					
All women (*n* = 10,757)	1979; 2001	+2.15 (+0.05, +4.30)	−1.39 (−1.96, −0.82)	+5.34 (+3.93, +6.77)	+1.19 (+0.56, +1.83)
0–19 years (*n* = 271)	No joinpoint detected	-	-	-	+1.32 (+0.41, +2.24)
20–39 years (*n* = 2,325)	2006	+0.70 (+0.30, +1.10)	+10.77 (+5.75, +16.04)	-	+2.27 (+1.46, +3.09)
40–59 years (*n* = 3,361)	2001	−0.98 (−1.67, −0.29)	+5.03 (+2.02, +8.13)	-	+0.66 (−0.27, +1.59)
60–79 years (*n* = 3,556)	1974; 2004	+9.58 (−1.34, +21.70)	−2.13 (−2.64, −1.62)	+6.90 (+3.71, +10.19)	+0.75 (−0.43, +1.94)
80+ years (*n* = 1,244)	1979; 1998	+2.14 (−2.33, +6.81)	−4.22 (−5.72, −2.70)	+0.71 (−1.35, +2.82)	−1.21 (−2.51, +0.11)

Time period 1970–2013, ICD-7 code 194 (http://www.socialstyrelsen.se/statistik/statistikdatabas/cancer)

*APC* annual percentage change (*APC 1* time from 1970 to first joinpoint, *APC 2* time from first joinpoint to 2013 or to second joinpoint, *APC 3* time from second joinpoint to 2013), *AAPC* average annual percentage change

**Fig. 1:**
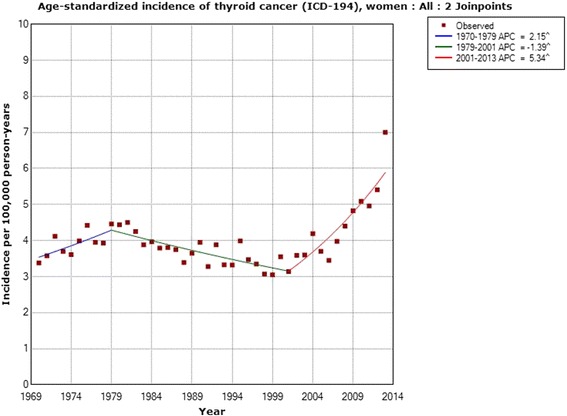
Joinpoint regression analysis of age-standardized incidence of thyroid cancer for women, all ages 1970–2013. Incidence per 100,000 inhabitants for ICD-7 code 194 according to the Swedish Cancer Register (http://www.socialstyrelsen.se/statistik/statistikdatabas/cancer)

**Fig. 2:**
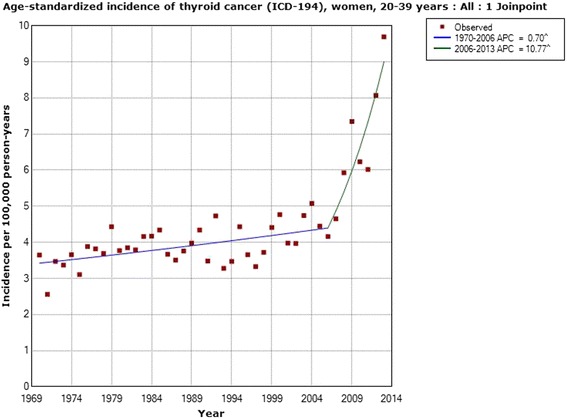
Joinpoint regression analysis of age-standardized incidence of thyroid cancer for women, aged 20–39 years 1970–2013. Incidence per 100,000 inhabitants for ICD-7 code 194 according to the Swedish Cancer Register (http://www.socialstyrelsen.se/statistik/statistikdatabas/cancer)

Figure 1 shows the joinpoint regression analysis of the age-standardized incidence of thyroid cancer (ICD-194) per 100,000 in all women during 1970–2013. A sharp increase is shown from 2001. For specific age groups, the highest APC was found in the age group of 20–39 during 2006–2013. Figure 2 shows the results for that age group with a joinpoint in 2006.

Table 2 shows the results for men. The incidence increased for all men during 1970–2013 with an AAPC of +0.77 % (95 % CI −0.03, +1.58 %). One joinpoint was detected in 2005 with a statistically significant increase in incidence during 2005–2013; APC +7.56 % (95 % CI +3.34, +11.96 %). Due to a low number of cases, no calculations could be made for subjects aged 0–19 years. In the age groups 20–39, 40–59 and 60–79 years the incidence increased for the whole period, although the AAPCs were not statistically significant. No joinpoint was found for ages 20–39 years. In the age group 40–59 years one joinpoint was found in 2006 with a statistically significant increase in incidence during 2006–2013; APC +9.92 % (95 % CI +1.92, +18.54 %). In subjects aged 60–79 years two joinpoints were found, 1980 and 2005. During 2005–2013 the APC was +8.41 % (95 % CI +4.02, +12.98 %). For men aged 80+ years the incidence decreased with a statistically significant AAPC and no joinpoint was found. These latter results were based on 390 cases.

**Table 2:** Joinpoint regression analysis of thyroid cancer incidence in men in the Swedish Cancer Register

ICD-7	Joinpoint location	APC 1 (95 % CI)	APC 2 (95 % CI)	APC 3 (95 % CI)	AAPC (95 % CI)
194					
All men (*n* = 4,234)	2005	−0.72 (−1.15, −0.29)	+7.56 (+3.34, +11.96)	-	+0.77 (−0.03, +1.58)
0–19 years (*n* = 76)	-	-	-	-	-
20–39 years (*n* = 651)	No joinpoint detected	-	-	-	+0.92 (−0.10, +1.94)
40–59 years (*n* = 1,271)	2006	−0.55 (−1.18, −0.09)	+9.92 (+1.92, +18.54)	-	+1.08 (−0.22, +2.41)
60–79 years (*n* = 1,846)	1980; 2005	+2.33 (−0.64, +5.40)	−2.14 (−2.91, −1.36)	+8.41 (+4.02, +12.98)	+0.79 (−0.31, +1.89)
80+ years (*n* = 390)	No joinpoint detected	-	-	-	−2.21 (−3.55, −0.85)

Time period 1970–2013, ICD-7 code 194 (http://www.socialstyrelsen.se/statistik/statistikdatabas/cancer)

*APC* annual percentage change (*APC 1* time from 1970 to first joinpoint, *APC 2* time from first joinpoint to 2013 or to second joinpoint, *APC 3* time from second joinpoint to 2013), *AAPC* average annual percentage change

**Fig. 3:**
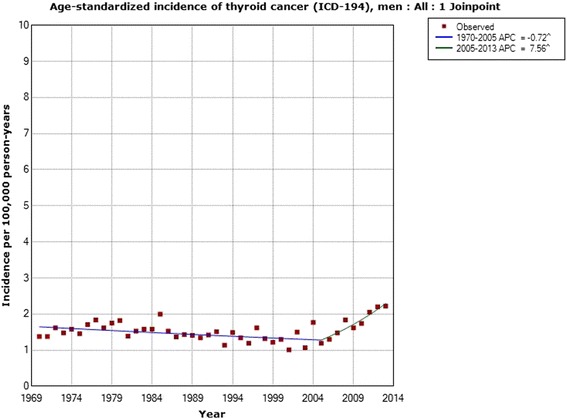
Joinpoint regression analysis of age-standardized incidence of thyroid cancer for men, all ages 1970–2013. Incidence per 100,000 inhabitants for ICD-7 code 194 according to the Swedish Cancer Register (http://www.socialstyrelsen.se/statistik/statistikdatabas/cancer)

**Fig. 4:**
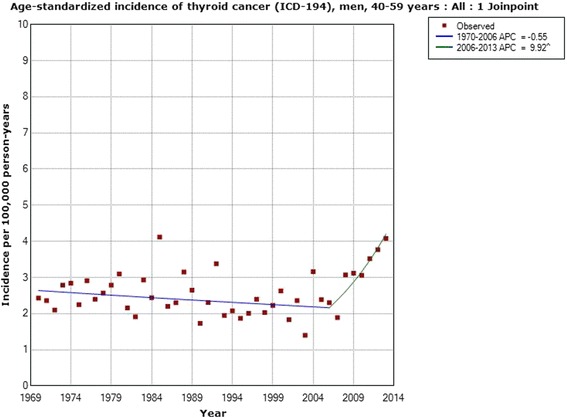
Joinpoint regression analysis of age-standardized incidence of thyroid cancer for men, aged 40–59 years 1970–2013. Incidence per 100,000 inhabitants for ICD-7 code 194 according to the Swedish Cancer Register (http://www.socialstyrelsen.se/statistik/statistikdatabas/cancer)

Figure 3 shows the joinpoint regression analysis of the age-standardized incidence of thyroid cancer (ICD-194) per 100,000 in men with an increasing incidence from 2005. Figure 4 shows the results for men aged 40–59 years with a joinpoint in 2006.

### Histopathological type

Trends in the age-standardized incidence for the time period 1993–2013 were calculated based on data from the Swedish Cancer Register. Due to no registered cases for some years for the anaplastic and medullary types no APC could be calculated. Incidence for the follicular type increased in women with +1.65 % (95 % CI −0.31, +3.64 %; *n* = 659), and in men with an APC of +0.40 % (95 % CI −2.26, +3.12 %; *n* = 281). No joinpoint was detected. The only statistically significant increase was found in the age group 0–39 years in women, APC +5.32 % (95 % CI +0.42, +10.46 %; *n* = 129). APC for mixed thyroid cancer was calculated in women to +2.52 % (95 % CI −0.62; +5.76 %; *n* = 232), and in men to +6.04 % (95 % CI +0.03, +12.41 %; *n* = 80). No joinpoint was detected. APC for different age groups could not be calculated since no cases were registered for certain years.

**Fig. 5:**
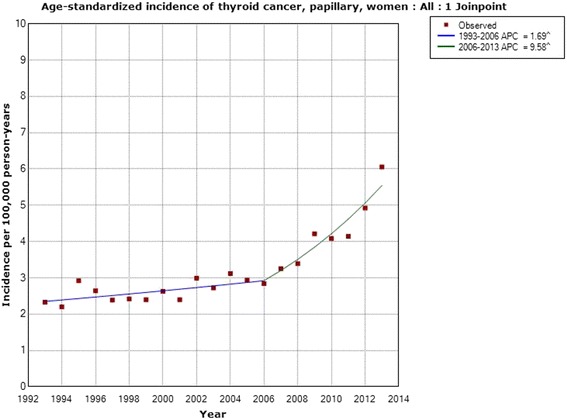
Joinpoint regression analysis of age-standardized incidence of papillary thyroid cancer for women, all ages, 1993–2013. Incidence per 100,000 inhabitants for ICD-7 code 194; data obtained from the Swedish Cancer Register

**Fig. 6:**
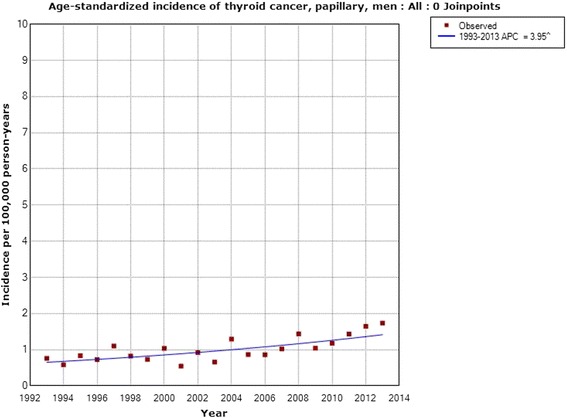
Joinpoint regression analysis of age-standardized incidence of papillary thyroid cancer for men, all ages, 1993–2013. Incidence per 100,000 inhabitants for ICD-7 code 194; data obtained from the Swedish Cancer Register

Regarding papillary thyroid cancer the incidence increased statistically significantly in women with an AAPC of +4.38 % (95 % CI +2.95, +5.84 %; *n* = 3,439). One joinpoint was detected in 2006; 1993–2006 APC +1.69 % (95 % CI +0.32, +3.08 %), 2006–2013 APC +9.58 % (95 % CI +5.85, +13.44 %), see Fig. 5. The incidence increased in men during 1993–2013 with an APC of +3.95 % (95 % CI +2.20, +5.73 %; *n* = 1,188). No joinpoint was detected, see Fig. 6. In the analyses of different age groups for women aged 0–39 years one joinpoint was detected in 2007; 1993–2007 APC +2.90 % (95 % CI +1.19, +4.64 %), 2007–2013 APC +11.11 % (95 % CI +4.59, +18.03 %), and in women aged 60–79 years in 2004; 1993–2004 APC −0.77 % (95 % CI −4.20, +2.78 %), 2004–2013 APC +9.16 % (95 % CI +4.08, +14.49 %). No joinpoint was detected in men in the analyses of different age groups.

### NORDCAN

**Fig. 7:**
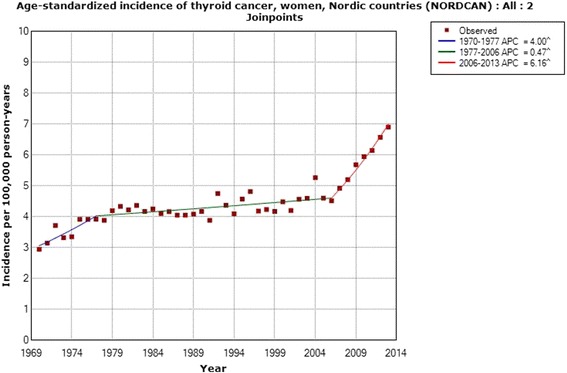
Joinpoint regression analysis of age-standardized incidence of thyroid cancer for women, all ages 1970–2013. Incidence per 100,000 inhabitants for ICD-10 code C73 in the Nordic countries according to NORDCAN (http://www-dep.iarc.fr/NORDCAN/english/frame.asp)

According to NORDCAN, the incidence increased statistically significantly in women during 1970–2013, Table 3. Two joinpoints were found, 1977 and 2006. Especially high APC was calculated during the time from the second joinpoint in 2006 to 2013; +6.16 % (95 % CI +3.94, +8.42 %). These results are displayed in Fig. 7 with a more than 2-fold increased incidence from 1970 to 2013.

**Table 3:** Joinpoint regression analysis of thyroid cancer incidence in women in the Nordic countries

ICD-10	Joinpoint location	APC 1 (95 % CI)	APC 2 (95 % CI)	APC 3 (95 % CI)	AAPC (95 % CI)
All women (*n* = 31,915)	1977; 2006	+4.00 (+1.83, +6.22)	+0.47 (+0.20, +0.73)	+6.16 (+3.94, +8.42)	+1.94 (+1.44, +2.45)

NORDCAN data, time period 1970–2013, ICD-10 code C73 (http://www-dep.iarc.fr/NORDCAN/english/frame.asp)

*APC* annual percentage change (*APC 1* time from 1970 to first joinpoint 1977, *APC 2* time from first joinpoint to second joinpoint 2006, *APC 3* time from second joinpoint to 2013), *AAPC* average annual percentage change

**Fig. 8:**
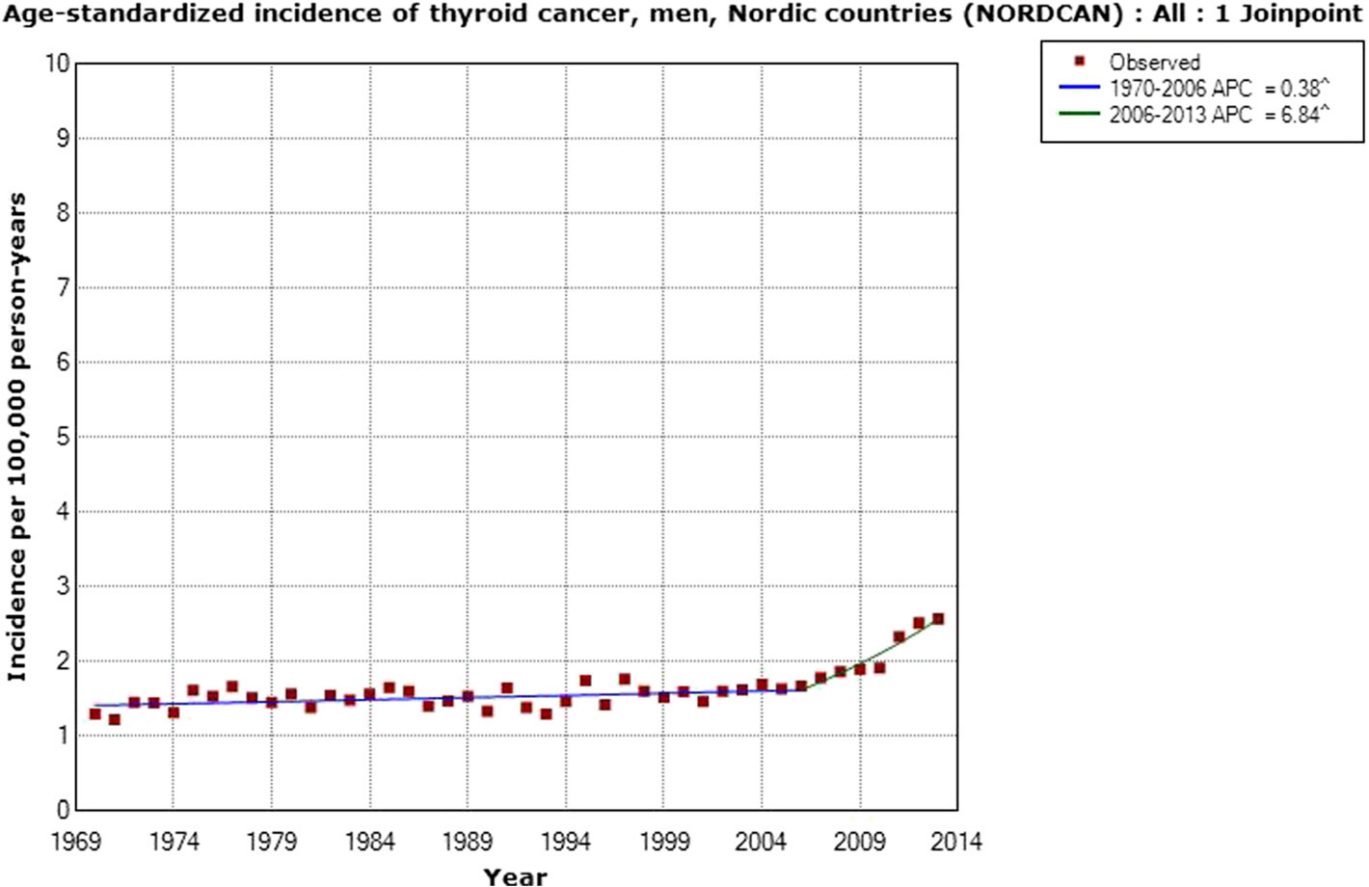
Joinpoint regression analysis of age-standardized incidence of thyroid cancer for men, all ages 1970–2013. Incidence per 100,000 inhabitants for ICD-10 code C73 in the Nordic countries according to NORDCAN (http://www-dep.iarc.fr/NORDCAN/english/frame.asp)

Also in men the incidence increased during 1970–2013 with an AAPC of +1.40 % (95 % CI +0.88, +1.93 %), Table 4. One joinpoint was detected in 2006 with an APC during 2006–2013 of +6.84 % (95 % CI +3.69, +10.08 %). As can be seen in Fig. 8, the incidence increased about 2-fold in men as well during the time period.

**Table 4:** Joinpoint regression analysis of thyroid cancer incidence in men in the Nordic countries

ICD-10	Joinpoint location	APC 1 (95 % CI)	APC 2 (95 % CI)	AAPC (95 % CI)
All men (*n* = 11,513)	2006	+0.38 (+0.12, +0.63)	+6.84 (+3.69, +10.08)	+1.40 (+0.88, +1.93)

NORDCAN data, time period 1970–2013, ICD-10 code C73 (http://www-dep.iarc.fr/NORDCAN/english/frame.asp)

*APC* annual percentage change (*APC 1* time from 1970 to joinpoint 2006, *APC 2* time from joinpoint to 2013), *AAPC* average annual percentage change

### Mobile phone calls

**Fig. 9:**
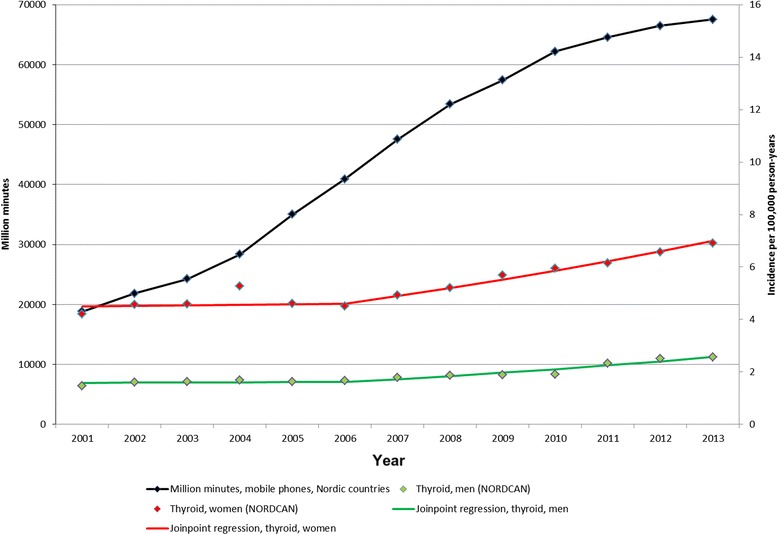
Number of out-going mobile phone minutes and incidence of thyroid cancer 2001–2013. Mobile phone minutes in millions in the Nordic countries (http://statistik.pts.se/PTSnordic/NordicBaltic2014/) and incidence per 100,000 person-years for all ages 2001–2013 according to NORDCAN (http://www-dep.iarc.fr/NORDCAN/english/frame.asp). Joinpoint regression analyses based on the time period 1970–2013

The number of total minutes of out-going mobile phone calls in million minutes is available for the Nordic countries for the time period 2001–2013 (PTS; http://statistik.pts.se/PTSnordic/NordicBaltic2014/). In Fig. 9 this data is shown in comparison with the joinpoint regression analysis of incidence of thyroid cancer in the Nordic countries for all ages during the same time period. Clearly, with a lag time of some years after the increasing number of out-going calls, the thyroid cancer incidence is increasing.

## Discussion

### Main results

The main finding of this register based study was an increasing incidence of thyroid cancer in Sweden during the whole study period 1970–2013 in both women and men, although not statistically significant in men. In both genders the incidence increased during the more recent study period, from 2001 in women and from 2005 in men. This increase was of similar magnitude and statistically significant for both groups.

Based on NORDCAN, we analyzed the thyroid cancer incidence during the same time period, 1970–2013, in the Nordic countries. A statistically significant increase in the incidence of thyroid cancer was seen throughout the whole time period. The same joinpoint, 2006, was found both for women and men. Interestingly, also the APC during 2006–2013 was of a similar magnitude in men and women. These results clearly show that the increasing incidence is not gender specific, meaning that women and men are equally affected and thus that the increase is caused by similar agent(s) for both genders.

We obtained data from the Swedish Cancer Register on different histopathology types of thyroid cancer for the time period 1993–2013. A statistically significant increase in incidence was found for papillary thyroid cancer, the type that is caused mainly by radiation [16]. The increase was seen in both men and women, in the latter with a joinpoint in 2006. The same joinpoint location was found for thyroid cancer incidence in NORDCAN in both men and women with a sharply increasing incidence from that year. We also found a statistically significant increase in incidence in men with mixed papillary thyroid cancer using the Swedish Cancer Register. These types are usually grouped together with the papillary variant, although the Swedish Cancer Register provided separate data and thus we could analyze these groups separately. Our results clearly indicate that the increasing incidence of thyroid cancer is mainly for the papillary type and may be caused by radiation. Both ionizing and non-ionizing radiation should be considered.

Just recently, statistics from the Swedish Cancer Register have been made official on all new cancer cases for 2014 [22]. For thyroid cancer there is a continuous increase in incidence in 2014 compared to 2013, by 12.1 % for men, (from 3.3 to 3.7), and by 11.2 % for women, (from 8.9 to 9.9; age standardized per 100,000 inhabitants).

### Towards understanding the increasing incidence

Thyroid cancer incidence is increasing in many countries. This has largely been restricted to small tumors of less than 2 cm with histopathological low aggressiveness in some studies [23]. Overall incidence rates increased during 1997–2008 in São Paulo, Brazil, especially the papillary variant that is the most radiosensitive type. It was concluded that the risk increase could not be only attributed to increased diagnostic procedures [24]. Increasing incidence, of especially the papillary type, was also reported from the Netherlands [25] and Canada [26]. Better access to healthcare and an increasing use of thyroid imaging causing ‘overdiagnosis’ has been suggested [27]. In a series of 2,654 patients that underwent FDG-PET/CT, 34 patients had incidental thyroid lesion, including 11 cancer cases [28]. In fact, it has been discussed that increasing diagnostic procedures may account for part of the increasing incidence of thyroid cancer, so called ‘overdiagnosis’, but a true increase cannot be excluded [27, 29].

A study of 18 cancer registers in the US showed an increased incidence of all thyroid cancers between 2000–2002 and 2010–2012 of 22.76 %. For papillary carcinoma of the thyroid, the incidence increased by 173.86 %. The increase included all sizes of papillary carcinoma, from those under one centimeter to those over 4 cm [30]. The incidence of thyroid cancer also increased during the study period 1997 through 2011 in Korea [31]. Papillary carcinoma showed the greatest increase with an APC of +25.1 % (95 % CI +22.7, +27.5 %) in men, and an APC of +23.7 % (95 % CI +22.9, +25.5 %) in women. It was concluded that the increase was partly a screening effect, but that among men born 1950 or later the exposure to risk factors may have changed. The steeply increasing incidence of thyroid cancer in Korea from early 2000 was also reported in other nationwide studies on cancer statistics [32, 33].

The impact of diagnostic changes during 2003–2007 on the rise in thyroid cancer incidence was studied in high-resource countries [29]. The study included the Nordic countries. It was postulated that diagnostic changes may account for ≥60 % of the cases in France, USA, Australia and the Republic of Korea, about 50 % in the Nordic countries and 30 % in Japan. It is noteworthy that the main increase in Sweden was found after that study period and thus cannot fully explain the results in our joinpoint analysis.

Increased exposure to thyroid-specific environmental carcinogens could be responsible, such as ionizing radiation (mostly medical radiation), increased iodine intake and chronic lymphocytic thyroiditis and environmental pollutants such as nitrates, heavy metals and other compounds largely used in the industrialized society [27]. Other factors that have been suggested include eating habits, smoking, living in volcanic areas, xenobiotics and viruses [34]. Certainly several of these factors are not relevant to Sweden, i.e., living in a volcanic area. Smoking is less common in Sweden now than previously [35] and there is no information on a sudden change in eating habits and exposure to xenobiotics and viruses. Increasing use of CT scans including of the thorax, head and neck might be of concern, especially since previous studies have shown an increased risk for thyroid cancer [36, 37].

### Ionizing radiation

Ionizing radiation is one established risk factor for thyroid cancer. Since the first correlation was reported in the late 1940s, several studies have confirmed the association. Especially studies of childhood X-ray treatment of thymus and scalp ringworm have established radiation as a risk factor, as well as among A-bomb survivors [37]. The dose-response curve seems to be linear and several studies have indicated that the risk increase begins between 5 to 10 years after irradiation. There seems to be a peak about 15–25 years post-irradiation, although the increased risk continues for a long time, and is probably life-long [38]. In fact, in Belarus and Ukraine an excess of thyroid cancer incidence was observed within 3 years after the Chernobyl accident in 1986 [39, 40]. Radioactive elements were released from the Fukushima Daiichi Nuclear Power Plant in March 2011. Using a latency period of up to 4 years an excess of thyroid cancer was reported in residents 18 years or younger [13]. The minimum empirical latency (induction time) has been reported to be 2.5 years in adults and 1 year for children for radiation induced thyroid cancer [41]. Of the 87 operated children in the Fukushima study papillary carcinoma of the thyroid was histologically confirmed in 83 [13]. Risk factors are younger age when exposed to radiation and female gender. In experimental studies, synergistic effects of radiation and chemicals that stimulate thyroid tissue proliferation have been clearly shown [42].

Of special concern nowadays is the thyroid radiation from CT medical examinations such as chest CT, whole body trauma CT etc. Increasing trends in the number of CT procedures in all Nordic countries were reported during 1993 to 2010 (https://www.stralsakerhetsmyndigheten.se/Global/Pressmeddelanden/2012/justification_statement_nordic_2012.pdf). The number per 1,000 of population increased from about 40 in the early 1990s to 100 or more at the end of the study period. It was concluded that CT procedures contribute currently to 50–80 % of the total population dose from medical X-ray. CT for pediatric use has increased and children are more sensitive to radiation compared to adults. Su et al. [36] concluded that especially chest CT-scans cause a high thyroid dose and contribute to the lifetime attributable risk of thyroid cancer.

Whole body PET-CT scanning is increasingly used in medicine. From 2006 to 2013 the number of examinations increased about 3 times in Sweden (http://www.skane.se/Upload/Webbplatser/RCC/PET-CT-150522.pdf). It was concluded that the examination is accomplished with substantial radiation dose and cancer risk including to the thyroid gland [43].

Dental radiography is widely used in dental care, both at the yearly to second yearly regular dental examination and when needed in more urgent visits. A case-control study from Kuwait showed a statistically significant dose response pattern with an increasing trend in risk for thyroid cancer with increasing numbers of dental x-rays. The association was essentially observed with papillary carcinoma [44]. Using lead collars or aprons during each dental x-ray can reduce the radiation dose, but these were not commonly used in the Kuwait study [44]. Another study showed that more than 10 dental x-rays increased the risk for thyroid cancer, especially the papillary type [45]. An increased risk of thyroid cancer has also been reported in female dentists and dental assistants [9]. It should be noted that these are retrospective studies. The radiation dose is nowadays lower for each investigation, but on the contrary dental x-ray investigations are more frequently used than previously.

### Radiofrequency radiation

One environmental factor that needs to be discussed in this context is the public’s increased exposure to the radiofrequency electromagnetic fields (RF-EMFs) due to the use of mobile and cordless phones. With the decreased subscription cost and innovations in technology, we have seen a large spread of mobile networking; mobile phones are not only used to make phone calls but also for using the internet. We have discussed that issue in relation to the increasing rate of brain tumors in the Swedish National Inpatient Register (IPR) and Causes of Death Register (CDR) [46]. Moreover, there has been a rapid increase in the use of wireless phones during the last two decades. An estimate of 6.9 billion mobile phone subscriptions worldwide was reported at the end of 2014 by the International Telecommunication Union [47]. Mobile phones were introduced in Sweden during the early 1980s, but the real increase of the use has taken place since the 1990s [48]. Desktop cordless phones have been used since the end of the 1980s. There are no official statistics on that use, but almost all desktop phones on the market are now of the wireless type. While used, wireless phones emit RF-EMFs.

The brain is the primary target for RF-EMFs during the use of wireless phones and an increased risk for brain tumors has been found in several studies (for overviews see [46, 49, 50]). The carcinogenic effect of RF-EMFs was evaluated at a meeting in May 2011 at the International Agency for Research on Cancer (IARC) at WHO in Lyon. The Working Group categorized RF-EMFs from mobile phones and from other devices that emit similar non-ionizing electromagnetic fields in the frequency range 30 kHz – 300 GHz as Group 2B, i.e., ‘possibly’ carcinogenic to humans [51, 52].

RF-EMF related organ-specific risk and exposure can be calculated. The personal RF-EMF exposure consists of far-field and near-field exposure. Far-field organ specific average (OSA) specific absorption rate (SAR) for RF-EMF exposure was calculated in a study by Lauer et al. [53]. The considered far-field sources were frequency modulation (FM) radio stations, television broadcasting stations, base stations for mobile phones and Digital Enhanced Cordless Telecommunications (DECT), and wireless fidelity (WiFi) hotspots. Besides the eye lens, skin and testis, the thyroid gland was the organ with the highest far-field OSA absorption. Also, near-field OSA absorption caused by mobile phone and cordless phone sources was calculated. The eye lens, skin and thyroid gland were the organs with the highest exposure other than the brain [53]. It can be summarized that both with near-field and far-field exposure, the thyroid gland was among the organs with the highest exposure. However, the near-field SAR calculations have several limitations [54]. Therefore, the mobile phone antenna location and distance to the thyroid gland need to be observed more closely.

The mobile phone handset antenna used to occupy a quarter of the mobile phone’s size. With the emergence of new communication protocols, new frequency bands (e.g. GSM, UMTS, and LTE) required incorporating more antennas into the mobile phone. This has in turn increased the space the antennas occupy. It must be noted that in order for the antenna to be efficient, a certain size is required. If an antenna size would be decreased, this would result in higher output power requirements, which would also expose the user to higher levels of radiation.

**Fig. 10:**
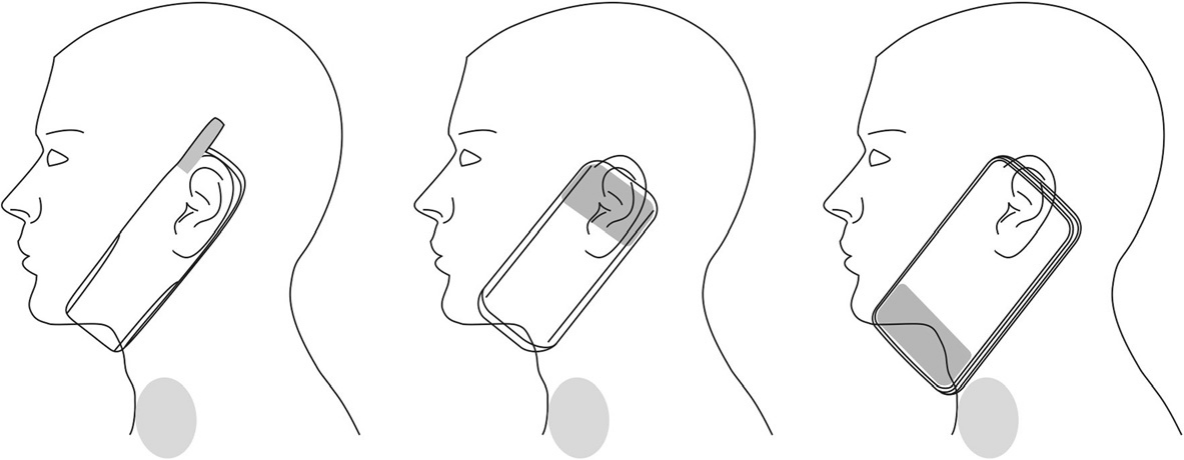
Mobile phone antenna placements in regard to the thyroid gland

As pictured in Fig. 10, three developments in the antenna design can be distinguished during the last two decades. The second generation (2G) mobile phones started in the 1990s with the external retractable monopole or helical antennas. The 2G GSM band operated at 800/900 MHz frequency band, later accompanied by 1,800 MHz band. Around the turn of the millennium, the external antennas were starting to disappear, replaced with new phone models with internal planar or microstrip antennas. The first internal antenna was introduced in 1998 and the first dual-band mobile phone, with the internal antenna, was introduced on the market in 1999 [55]. The internal antennas were positioned at the top of the telephone. With the emergence of the smartphones in the mid and late 2000s, the internal antenna location started to shift from the top of the phone to the bottom. Currently, the majority of smartphone models have their antenna positioned at the bottom of the phone.

During the last decade, the smartphone popularity has surpassed both the laptop and desktop computer sales in many world markets. Smartphone sales have risen dramatically and replaced the basic feature phone market [56]. 3G and 4G have become the new standards for mobile communications in mature markets. Since the launch of these new protocols the mobile data consumption has been increasing, of which most takes place on smartphones [57].

Compared to mobile feature phones, smartphones potentially provide higher RF-EMF exposure. Smartphones can connect to several networks: they come with built-in communication protocols for 2G, 3G, 4G long-term evolution (LTE), WiFi and Bluetooth for short distance and near field communications (NFC).

**Fig. 11:**
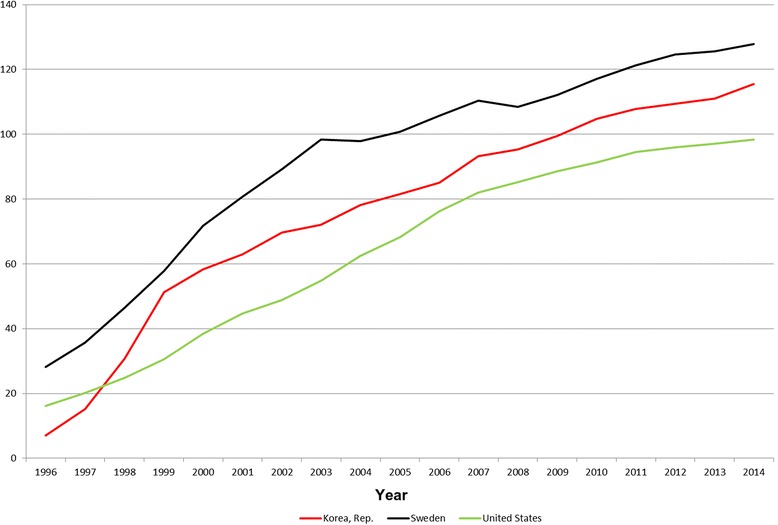
Mobile phone subscriptions per 100 persons in South Korea, Sweden and United States from 1996–2014 [58]

Figure 11 provides mobile phone subscription trend from 1996 to 2014 for Sweden, USA and South Korea [58]. The World Bank’s indicator includes the number of postpaid subscriptions, and the number of active prepaid accounts; the indicator applies to all mobile cellular subscriptions that offer voice communications [58].

Certainly, thyroid gland exposure to RF-EMFs has increased, especially during the last one or two decades. It should be further investigated as a cause of the increasing incidence of thyroid cancer in the Nordic countries as well as several other countries. The short induction time for thyroid cancer after exposure to ionizing radiation, as discussed above, should also be considered in that context.

### RF-EMFs, thyroid hormones and morphology

Radiofrequency radiation at 2.45 GHz at a non-thermal level modified the morphology of the thyroid gland. The central and peripheral follicles presented increased size and the thickness of peripheral septa decreased. Peripheral follicles increased in size with repeated exposure at 3 W power [59].

In another study on rats, whole body exposure to 900 MHz pulse-modulated RF radiation that was similar to that emitted by the global system for mobile communications (GSM) mobile phones caused pathological changes in the thyroid gland. The gland structure was altered and caspase-dependent pathways of apoptosis were enhanced [60].

Thyrocytes were exposed to RF-EMFs at mobile phone frequency for 3 h in a study on human thyroid cells. This led to increased proliferation of thyrocytes (*p* = 0.007). It was concluded that a proliferative effect was seen that may link RF-EMFs with carcinogenesis [61]. The same research group used a signal generator exposing the human thyroid primary cells at continuous wave (CW) 900 MHz with maximum SAR of 0.170 W/kg. They failed to find an association between this exposure and a carcinogenic effect on thyroid cells [62].

Detrimental effects on the thyroid function with altered levels of thyroid hormones in humans exposed to RF-EMF from mobile phones [63], and also including base stations [64] has been reported. In rats exposed to 900 MHz RF-EMF [65] and 2,450 MHz RF-EMF altered thyroid levels were found [66]. Alternations of thyroid stimulating hormone (TSH) levels were seen in one of the rat studies with statistically significant decreased levels [65], whereas the change was not statistically significant in the other rat study [66]. In humans there was a statistically significant increase of the TSH level following mobile phone use [63]. Elevated levels of TSH in humans is associated with thyroid growth and has been postulated to increase the risk for thyroid cancer [67, 68]. Certainly thyroid function deserves to be further studied in person using wireless phones.

### Strengths and limitations

Our study was based on official register data. The completeness of registration of all cancer cases in the Swedish Cancer Register has been questioned [69]. The underreporting to the register was mainly related to a lack of cytology and histopathology for cancer diagnosis. Especially visceral tumors were underreported in a 2009 study [70]. In another study by investigating pancreatic and biliary tract cancer, a large underreporting to the Swedish Cancer Register for the years 1990–2009 was found [71]. Unfortunately there exists no specific study about the completeness of thyroid cancer registration.

The thyroid gland is easily accessed for biopsy. Benign nodules are not reported to the Cancer Register, and for confident diagnosis, cytology and/or histopathology is needed. Based on the information published in “Cancer Incidence in Sweden”, all thyroid cancer diagnoses (100 %) were based on microscopic confirmations during our study period, with a few exceptions when it was based on 99 % microscopy.

In an ecological study there is no individual exposure data set. Thus a causal association with any of the suggested exposure types cannot be established. The results of this paper suggest that further research is required.

## Conclusions

This study has shown an increasing incidence of thyroid cancer in Sweden and the Nordic countries. Better diagnostic imaging cannot solely account for the increase. Increased use of CT and PET-CT for medical examinations has elevated the population’s exposure to the ionizing radiation and should be considered as a risk factor. Exposure to RF-EMFs also merits in-depth investigation. The design of our study does not permit conclusions regarding causality.

## Abbreviations

AAPC, average annual percentage change APC, annual percentage change; CDR, Causes of Death Register CI, confidence interval CT, computed tomography CW, continuous wave DECT, Digital Enhanced Cordless Telecommunications; FM, frequency modulation IARC, International Agency for Research on Cancer; IPR, Swedish National Inpatient Register LTE, long-term evolution; MRI, magnetic resonance imaging NFC, near field communications; OSA, organ specific average RF-EMF, radiofrequency electromagnetic fields; SAR, specific absorption rate TSH, thyroid stimulating hormone; WiFi, wireless fidelity
